# How can the utilisation of help for mental disorders be improved? A quasi-experimental online study on the changeability of stigmatising attitudes and intermediate variables in the process of utilisation

**DOI:** 10.1186/s12889-021-12125-5

**Published:** 2021-11-19

**Authors:** Thomas McLaren, Lina-Jolien Peter, Samuel Tomczyk, Holger Muehlan, Susanne Stolzenburg, Georg Schomerus, Silke Schmidt

**Affiliations:** 1 Department of Health and Prevention, Institute of Psychology, University of Greifswald, Greifswald, Germany; 2 Department of Psychiatry and Psychotherapy, Medical Faculty, University Leipzig, Leipzig, Germany; 3 Department of Psychiatry, University Medicine of Greifswald, Greifswald, Germany; 4 Department of Psychiatry and Psychotherapy, University of Leipzig Medical Center, Leipzig, Germany

**Keywords:** Mental health, Help-seeking behaviour, Depression, Continuum belief of mental illness, Causal beliefs, Mental health literacy, Self-efficacy, Anti-stigma intervention, Quasi-experimental online-study

## Abstract

**Background:**

Epidemiological studies show that even in highly developed countries many people with depression do not seek help for their mental health issues, despite promising prevention approaches encouraging people to seek help and reduce self-stigma. Therefore, an anti-stigma intervention study to support help-seeking behaviour will be developed on the basis of the newly explicated “Seeking Mental Health Care Model”.

**Methods:**

A quasi-experimental online study will be carried out to assess the effect of different intervention variables relevant for the help-seeking process. The study is conceived as a fractional factorial design. Participants will be screened for depressive complaints (PHQ-9 sum score ≥ 8) and current psychiatric/psychotherapeutic treatment. After baseline assessment the participants will be randomly allocated into one of the 24 study groups receiving different combinations of the vignette-based intervention aiming to reduce stigma and support help-seeking. Next, relevant outcome measures will be administered a second time. In a 3- and 6-month follow-up help-seeking behaviour will be measured. Gamified elements and avatar-choice techniques will be used to heighten study immersion and adherence.

**Discussion:**

On the basis of the project results, promising research and intervention perspectives can be developed. Results, firstly, allow for a more detailed empirical investigation and conceptualisation of the stages of mental health care utilisation, as well as an examination of theoretical approaches to stigmatisation. Secondly, our online study could provide insights for an evidence-based design and evaluation of online interventions for people with a mental illness.

**Trial registration:**

German Clinical Trials Register: DRKS00023557. Registered 11 December 2020. World Health Organization, Universal Trial Number: U1111–1264-9954. Registered 16 February 2021.

**Supplementary Information:**

The online version contains supplementary material available at 10.1186/s12889-021-12125-5.

## Background

Epidemiological studies show that even in countries with well-developed primary care and specialised mental health services most people with a mental illness do not seek treatment for their disorders, or only after considerable delay [6, 63]. In Germany two thirds of people with depressive symptoms do not seek mental health care [44]. This gap between health care need and utilisation exists despite modern developments in health promotion as well as selective preventive efforts such as mental health awareness campaigns. Moreover, people are systematically labelled and stigmatised for their mental illness [11, 38, 45]. People with a mental illness are often seen as dangerous, unpredictable, unreliable, and not normal [3]. The societal stigmatisation of people with a mental illness can be seen on many different levels, whether it is in personal day to day interactions, in working environments, on an institutional level, or in the media [11, 29]. A person with a mental illness who is aware of, agrees with, and internalises publicly endorsed stigmatising stereotypes, self-stigmatises and can experience loss of self-esteem, shame, and helplessness, inhibiting help-seeking behaviour [11, 47]. Therefore, it is important to address stigma in interventions supporting health care utilisation.

In a previous project the direct impact of stigmatising attitudes on help-seeking behaviour was examined. Results showed that stigmatising attitudes were negatively associated with a perceived need for help [59] and the use of professional psychiatric and psychotherapeutic help (β = − 0.19, *p* = 0.08). Furthermore, the main predictor for health care utilisation was the intention to seek help, which explained 22–37% of the variance of actual help-seeking behaviour and was especially strong for seeking psychotherapeutic help (β = 0.31, *p* < 0.001 [60];). Additionally, further findings concerning the self-identification as mentally ill [57], as well as a subjective sense of illness [58] and their respective association with help-seeking intention and behaviour were considered. On the basis of these findings it became evident that the working model had to be extended by one superordinate and five intermediary variables to account for the complex ways in which stigma impacts the help-seeking process. We use this extended and newly explicated model as the theoretical foundation of our present study.

**Fig. 1:**
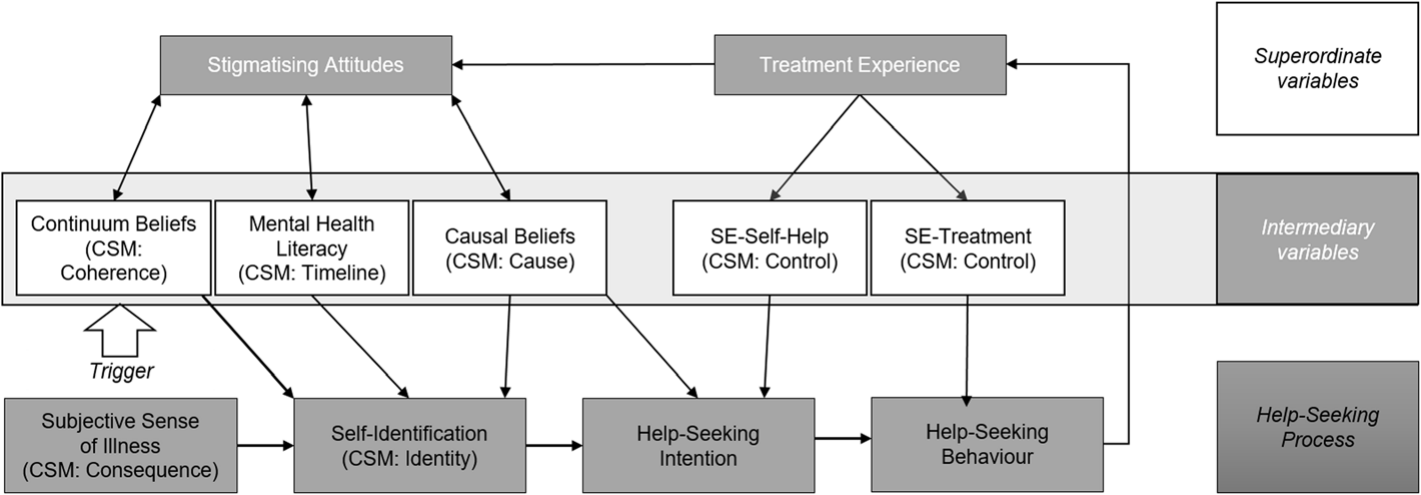
Seeking Mental Health-Care Model. Note. This figure shows the process in which a person seeks out mental health-care, starting with a person becoming aware of their own psychological complaints. CSM = Common-Sense Model of Self-Regulation, parallelised cognitive illness representations. SE = Self-Efficacy

According to the “Seeking Mental Health Care Model” (see Fig. 1) a person moves from becoming aware of their own psychological complaints, self-identifying as mentally ill, forming the intention to seek help (i.e., accepting that one needs help), to eventually seeking out professional help. On the superordinate level previous treatment experience [57] is added to stigmatising attitudes, as both influence the whole help-seeking process through their impact on the intermediary variables as well as their direct association with help-seeking behaviour. On an intermediary level, the following five variables are identified to have an impact on the help-seeking process and stigmatising attitudes respectively. *Continuum belief of mental illness*, the belief that there is a fluid transition between health and illness is associated with less stigmatising attitudes (e.g., [50]). *Mental health literacy*, the beliefs, attitudes, and knowledge concerning mental illness and the treatment thereof [25] is included, as studies point to its positive impact on the help-seeking process (e.g., [26, 53]). *Causal beliefs,* the subjective belief of the causes of the symptoms the person is experiencing is included. Studies show differential results of the effects of causal beliefs on stigma and help-seeking (e.g., [33, 57]). Finally, self-efficacy is included in the model, which we again specified as the (4) *self-efficacy to help oneself* and (5) *self-efficacy to seek out professional* help, since they seem to have a different effect on help-seeking intention and behaviour [60, 61]. The Common-Sense-Model of Self-Regulation (CSM [36];) is used as a corresponding theoretical framework. The CSM describes the subjective management of health threats, that is, how a person labels their symptoms (identity), rates the onset, duration, and fluctuation of them (timeline), attributes causal antecedents to their symptoms (cause), evaluates their perceived impact (consequence), treats the symptoms themselves (perceived control), and comprehends them (coherence). These six cognitive illness representations, which have been shown to have an impact on illness outcomes [21], are parallelised with the corresponding intermediary variables of our proposed model (see Fig. 1) to integrate the separate constructs into one holistic model (e.g., *coherence* is parallelised with the construct *continuum belief of mental illness*).

### Research agenda of the current study (stigma-II)

The present study will examine the effects of a short, informational, one-time administered intervention, aimed at influencing the intermediary process variables (i.e., continuum belief of mental illness, mental health literacy, causal beliefs, and self-efficacy to help oneself as well as seeking professional help) which in turn will reduce stigmatising attitudes and facilitate help-seeking.

The study will focus on people with at least mild depressive complaints, who are currently not receiving professional psychiatric/psychotherapeutic treatment, and live in Germany. Possible effects of the Covid-19 pandemic are assessed (e.g., adherence to regulations, mental-health burden through regulations), since it is assumed that the pandemic and lockdown-regulations had an impact on psychological well-being in general [14].

Participants of the experimental groups will receive a text-based vignette followed by a combination of online-administered interventional vignettes (either as a text and/or a video) designed to influence the five intermediary process variables described above. The participants of the control group will only be presented with the case-vignette. The primary and secondary outcomes will be measured directly pre- and post-intervention, as well as after 3 and 6 months in subsequent follow-up assessments. The primary outcome “help-seeking behaviour” will be assessed at the subsequent follow-ups. The study is designed as a quasi-experimental online study and the chosen design of experiment is the fractional factorial design [15] to adequately account for the complexity of all the possible intervention-combinations required to answer the research questions.

Prospectively, the study’s results will lead to a better understanding of the complex interplay between stigmatising attitudes and mental health care utilisation and which intermediary mechanisms play a pivotal role therewith. Moreover, the results could be used to design population wide prevention campaigns to encourage help-seeking when suffering from depressive complaints, on the one hand, and reduce stigmatising attitudes, on the other hand.

#### Primary research questions

The intervention is expected to directly and indirectly influence help-seeking intention and behaviour positively, resulting in a higher intention as well as higher rates of professional health care utilisation. Our primary research questions are:

1. Does a biopsychosocial causal model of depression make it easier to articulate an intention to seek health care treatment?
  H1: The agreement with a biopsychosocial- vs. a mono-causal model leads to a higher help-seeking intention.
2. Does the strengthening of context-specific self-efficacy (self-efficacy to help oneself and to seek professional help) influence treatment intention or utilisation?
  H2: Enhancing self-efficacy to help oneself leads to less intention to seek professional help (H2a), whereas enhancing self-efficacy to seek professional help leads to higher help-seeking intention (H2b) and help-seeking behaviour (H2c).
3. What importance does previous treatment experience have for the development of both self-efficacies and the last stages of the process of help-seeking?
  H3: Positive treatment experience is associated positively with both self-efficacies (H3a), help-seeking intention (H3b) and behaviour (H3c).

#### Secondary research questions

The intervention is expected to influence different stigmatising attitudes and self-identification as mentally ill. Our secondary research questions are:

4. Does a conception of depression as a manifestation on a mental health-illness continuum favour the readiness for self-identification as mentally ill? Does it also have a reducing effect on stigmatising attitudes?
  H4: The agreement with continuum beliefs leads to higher self-identification (H4a) and reduces stigmatising attitudes (H4b).
5. Does the strengthening of mental health literacy have a reducing effect on stigmatising attitudes and favour the readiness for self-identification as mentally ill?
  H5: The strengthening of mental health literacy leads to a higher self-identification (H5a) and reduces stigmatising attitudes (H5b).
6. Can a biopsychosocial causal model of depression have a reducing effect on stigmatising attitudes? Does it also favour the readiness for self-identification as mentally ill?
  H6: The agreement with a biopsychosocial causal model leads to a higher self-identification (H6a) and reduces stigmatising attitudes (H6b).
7. What importance does previous treatment experience have for the readiness for self-identification as mentally ill and the expression of stigmatising attitudes?
  H7: Positive treatment experience is associated positively with self-identification (H7a) and negatively with stigmatising attitudes (H7b).

#### Explorative research questions

In addition, open research questions pertain to how these intermediary variables interact with each other and, furthermore, influence help-seeking intention and behaviour as well as stigmatising attitudes, respectively. Also, the role of acute symptom severity on the whole process is unclear. Since no theoretically derived interaction could be hypothesised, the analyses will be explorative.

## Methods/design

A fractional factorial design is chosen, due to the number of influenced variables and the complexity of the study. This type of design will allow for an economic assessment, reducing the number of groups needed while ensuring that sufficient data for the primary and secondary outcomes can be collected. The conditions are systematically varied to determine the necessary within group condition-combinations after calculating the number of groups needed by multiplying the cartesian products of the variables with two and three levels, respectively: x = 3^2^ * 2^3^ = 72 (i.e., full factorial design). Fractioning the design by subtracting a variable-factor from the three-level variable resulted in our design (see Table 1) with a resolution of V and, therefore, being able to differentiate between main effects and two- (three-) way interactional effects: x = 3^2–1^ * 2^3^ = 24. For more information on fractional factorial designs refer to Dean et al. [15] and Siebertz, van Bebber, and Hochkirchen [55].

**Table 1:** Fractional factorial design with 24 groups

Experimental group	Intervention content				
	Continuum belief	Mental health literacy	Causal belief	Self-efficacy (self-help)	Self-efficacy (treatment)
1 (control group)					
2				T	T
3		T			T
4		T		T	V
5			T		T
6			T	T	V
7		T	T		V
8		T	T	T	
9	T				T
10	T			T	V
11	T	T			V
12	T	T		T	
13	T		T		V
14	T		T	T	
15	T	T	T		
16	T	T	T	T	T
17	V				V
18	V			T	
19	V	T			
20	V	T		T	T
21	V		T		
22	V		T	T	T
23	V	T	T		T
24	V	T	T	T	V

*Note.* T = text-based intervention, V = video-based intervention. Information for both intervention-types are the same. The groups are determined through a priori systematic variation and fractioning of the condition-combinations. The design has a resolution of V and can statistically differentiate between main and two- (three-) way interactional effects

The first part of the study will be separated into two different assessment times, both taking about 25 min to be administered, allowing for a collection of pre- and post- intervention data. During the baseline assessment participants will answer questionnaires and will be asked to participate in the second part of the study, to which they will receive an e-mail invitation 36 h later. In the second part of the study all participants will receive the online intervention (see Intervention), after which the post-assessment is administered. The participants will be informed that they will be receiving an invitation via e-mail to participate in the three- and six-month follow-up assessment.

### Study information

#### Power & sample size

**Fig. 2:**
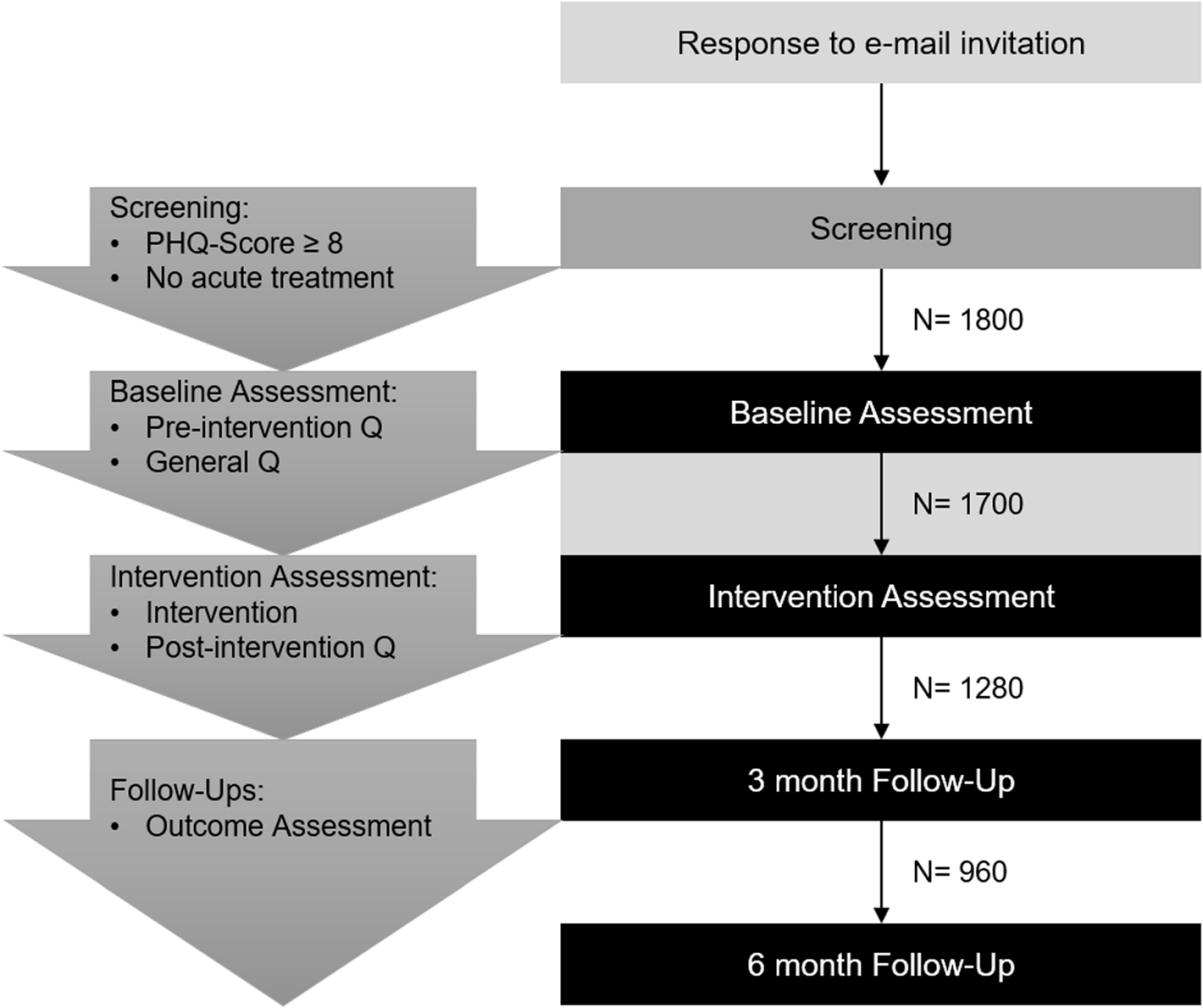
Study procedure and participant flow. Note. This figure shows the study procedure and the participant flow with the desired sample sizes from one assessment point to the next (estimated N). Q = questionnaire

The required sample size is based on calculations for ANOVA (repeated measures, within-between interaction) expecting small effect sizes of *f* = 0.10 due to the limited intervention administration time and the online setting. A significance level of α = 0.05 and a power of *1-*β = 0.95, as well as the a priori determined 24 groups were included in the calculation. The free software G*Power [16] was used to calculate the required sample size of *N* = 840. This would mean *n* = 35 in each group. To stray on the side of safety we strive to recruit *n* = 40 for each group, a total of *N* = 960 participants, to complete the second follow-up after 6 months of intervention administration. Based on previous experience we estimate a drop-out rate of 47% between the first assessment time and the second follow-up 6 months later, we aim to recruit *N* = 1.800 at the beginning of the study (see Fig. 2).

#### Inclusion criteria

Participants will be included if they are 18 years and older and score ≥ 8 on the PHQ-9, indicating at least mild depressive complaints [31]. This cut-off score was decided upon after extensive research on the use of the PHQ in comparable research [30, 37]. The participants have to be able to read and comprehend German.

#### Exclusion criteria

Participants will be excluded if they are currently receiving psychiatric or psychotherapeutic treatment. There are no further exclusion criteria.

#### Recruitment

Participants will be invited by the online-panel “respondi AG” (ISO 20252 certified). At the first recruitment time (January/February 2021), “respondi AG” has access to approx. 80.000 active panellists via the Mingle online-access panel, whereby general representativity is controlled for through quotas. An active panellist can be anyone who is registered in a double-opt-in process and has filled out general questionnaires as well as participated in at least one survey the past 3 months, showing that they are cognitively capable of participating in online studies. No further restrictions are imposed by “respondi AG”. It is feasible that the required case number will be reached.

Participants will receive comprehensive written information about the study, after which they can give electronic informed consent. The participants will be screened according to the inclusion and exclusion criteria. Quotas will be set in place concerning gender and age-groups. Approximately 35% of the study population should be male to represent the gender distribution prevalent amongst people with depression and the age groups should be distributed as equally as possible, corresponding to the German demographic structure. These quotas will be placed by the online-panel as well as within the questionnaire algorithm.

Participants who complete the baseline and intervention assessments will be invited to the subsequent follow-ups 3 and 6 months later via e-mail. Incentive will be provided through the online-panel at each successive assessment time, respectively.

#### Allocation & participation timeline

The experiment-platform Unipark (https://www.unipark.com) will automatically manage allocation and each participant will be placed randomly into one of the 24 groups following an internal algorithm. Figure 2 shows the participant flow including the desired sample size at each of the four assessment times.

### Intervention

The intervention material was produced after each intermediary variable was extensively researched and refined successively after thorough review by a team of four experts. Based on the material two videos were created using the online-software Powtoon (https://www.powtoon.com/). The videos are based on the text material of the variables (1) continuum belief of mental illness and (5) self-efficacy to seek out professional help.

A first-person narrative style was chosen, because stigmatising attitudes are changed more effectively when people come into direct contact with a person suffering from depression instead of generally reading about them [13]. Furthermore, the avatar-choice technique was implemented for better identification with the intervention content [17].

During the intervention, the avatars will firstly introduce themselves and the problems they are having, self-labelling the issues as a depressive episode. Secondly, a random combination of up to five texts or videos are presented to the participants (combinations are shown in Table 1). Afterwards, a question prompts the participant to reflect on the information presented by the avatar and assess, whether they agree with the information and would act similarly to the avatar. Using this method, the avatar narrates their story, and in so doing imparts information concerning the intermediary process variables. This type of gamified intervention design was decided upon after researching ways in which people are motivated to take part in longer online experiments [19]. An avatar freely chosen and who imparts their knowledge in a comprehensive story-line narrative is important for immersion [54] and in general motivates higher study participation [17]. Also, there is evidence suggesting that both older [28] and younger adults [54] benefit from gamified interventions and that gamified e-health interventions are effective in both mental health [46] and health-related behaviour [24] contexts.

The intervention material was evaluated during cognitive debriefings [35] with 15 people suffering from depressive complaints, of whom ten had previous treatment experience. For higher standardisation a structured interview guideline was written and the interviewers instructed beforehand. The interview transcripts were used to adapt the intervention material accordingly. The intervention structure and material can be seen in the supplement to this paper*.*

### Outcome measures

Table 2 shows the variables that will be investigated at different times during the study and the instruments used to assess these. They have been classified into general psychological, intermediary process, help-seeking, stigmatisation, and context assessment measures. The final selection of the instruments, some adapted to fit the current research setting, were based on theoretical research and pre-study test results (*N* = 227), in which psychometric properties, especially of the adapted instruments, were analysed.

**Table 2:** Questionnaires employed at the different study assessment times

Study assessments & measures	Number of items	Study time points			
		T0	T1	T2	T3
Socio-demographic					
Gender	1	X			
Month& year of birth	2	X			
Family status & partner	3	X			
Education	2	X			
Employment	2	X			
Income	1	X			
General psychological					
Subjective general health (SF-1)	1	X		X	X
Quality of life (EUROHIS-QoL Index)	8		X	X	X
Self-construal Scale (SCS)	24	X			
Subjective Socio-Economic Status	1				X
Intermediary process					
Continuum beliefs (CB_new)	12	X	X	X	X
Causal beliefs (List)	18	X	X	X	X
Mental health literacy, depression specific (D-Lit)	12	X	X	X	X
Self-efficacy to help oneself	6	X	X	X	X
Self-efficacy to seek out professional help	7	X	X	X	X
Help-seeking					
Subjective sense of illness (B-IPQ-R)	9	X		X	X
Self-identification as mentally ill (SELF-I)	5	X	X	X	X
Attitudes towards help-seeking (ATSPPH-SF)	10	X	X	X	X
Motivation behind help-seeking (ACMQ)	6	X	X		
Help-seeking intention (List)	16	X	X	X	X
Help-seeking subjective norms, attitudes and perceived control (TPB)	10	X	X	X	X
Help-seeking behaviour/ health-care utilisation	18			X	X
Stigmatisation					
Perceived public stigma and the agreement with stigma (SSMIS-public & -self-SF)	10	X	X	X	X
Social distance stigma (SDS)	7	X	X	X	X
Discrimination of mental illness	3	X	X	X	X
Sense of blame for mental illness	4	X	X	X	X
Self-stigma for seeking out professional help (SSOSH-SF)	3	X	X	X	X
Shame for being mentally ill and seeking out help	2	X	X	X	X
Emotional reaction towards a person with mental illness (ERMIS)	10	X	X	X	X
Context					
Depressive Symptoms (PHQ-9)	9	X	X	X	X
Anxiety Symptoms (PHQ-anxiety module)	7		X		
Differential diagnosis	1		X		
Previous treatment experience	3–7		X		
Perceived availability and accessibility to the health care system	1–5		X		
Covid-19 questions	4	X		X	X
	∑ = 246				

*Note.* T0 = baseline assessment, T1 = intervention assessment, T2 = follow-up after 3 months, and T3 = follow-up after 6 months. Intervention was administered online during T1

#### General psychological measures

With the *SF-1* single item measure for self-reported health status (SRHS [39];), included in the SF-36 [40], participants are asked the question: “In general, would you say your health is?” with the response options 1 = “poor”, 2 = “fair”, 3 = “good”, 4 = “very good”, and 5 = “excellent”.

With the *EUROHIS-QoL Index* [5] general quality of life is assessed with a reliability score of α = .85. Different response options are given, depending on the question (e.g., “How would you describe your quality of life?”).

With the *Self-Construal Scale* [62] four context-relevant self-construals are assessed (Making decisions, Looking after oneself, Communicating with others, Dealing with conflicting interests) with reliability scores between α = .74–.87. Participants are asked how well a statement describes them (e.g., “Usually I follow the advice of others when making important decisions”) on a 5-point Likert scale from 1 = “doesn’t describe me at all” to 5 = “describes me very well”.

With the German version of the *MacArthur Scale* [22] the subjective socio-economic status is assessed. The participants are asked to define their status on a scale from 1 = “lowest status” to 10 = “highest status” after being asked to imagine that all people in Germany are somewhere on a “social ladder”, the people at the top having the highest income, best education, and best jobs.

#### Intermediary process measures

With a new scale, taken and adapted from different measurements (considering the meta-analysis of [42]) continuum belief of mental illness is assessed. Participants are asked whether they agree with the statements (e.g., “Now and again most of us have symptoms of a mental illness”) on a 5-point Likert scale from 1 = “don’t agree at all” to 5 = “I agree completely”. Final reliability scores will be calculated with the data. This instrument will be used to test the hypothesis H4.

With a short form of the *Depression Literacy Scale* [20] depression literacy is assessed. Participants are asked whether a statement (e.g., “Too little or too much sleep can be a sign of depression”) is 1 = “true”, 2 = “false”, or 3 = “don’t know”. Based on secondary data analysis, the reliability score for the short-form is α = .72. This instrument will be used to test the hypothesis H5.

With a list of possible causes [51] causal belief is assessed. Participants are asked whether they believe a listed cause (e.g., “Living in a big city”) is a cause for their complaints on a 5-point Likert scale from 1 = “definitely is not a cause” to 5 = “definitely is a cause”. This instrument will be used to test the hypotheses H1 and H6.

Self-efficacy is assessed with context-relevant adaptations of items taken from the BRAHMS-Study [43] and a study assessing the health care use of homeless people [23]. Concerning self-efficacy for self-help, participants are asked to rate how certain they are that they can overcome specific barriers (e.g., “… if my complaints don’t get better despite my efforts”) on a 5-point Likert scale from 1 = “very uncertain” to 5 = “very certain”. Concerning self-efficacy for seeking professional help, participants are asked to rate how confidant they are that they can overcome barriers of the health care system (e.g., “I am able to deal with long waiting times”) on a 5-point Likert scale from 1 = “not confident” to 5 = “very confident”. Final reliability scores for both of these adapted instruments will be calculated with the data. These instruments will be used to test the hypotheses H2 and H3.

#### Help seeking measures

With the *B-IPQ-R* [8] the subjective sense of illness is assessed. All of the items except the causal question are rated using a 0 to 10 response scale. Five of the items assess cognitive illness representations: consequences (item 1), timeline (item 2), personal control (item 3), treatment control (item 4), and identity (item 5). Two of the items assess emotional representations: concern (item 6) and emotions (item 8). One item assesses illness comprehensibility (item 7). Assessment of the causal representation is by an open-ended response item adapted from the IPQ-R, which asks patients to list the three most important causal factors in their illness (item 9). Test-Retest reliability after 6 weeks lies between .42 (personal control) and .75 (identity).

With the *SELF-I* [48, 52] the self-identification as mentally ill is assessed. Participants are asked to appraise their current problems (e.g., “My present problems could be the first signs of a mental disorder”) on a 5-point Likert scale from 1 = “don’t agree at all” to 5 = “agree completely”. The scale has excellent internal reliability (α = .90). This instrument will be used to test the hypotheses H4, H5, H6, and H7.

With the *ATSPPH-SF* [18] attitudes towards help-seeking is assessed. Participants are asked to rate how much they agree with certain statements regarding seeking help from a psychiatric or mental health professionals (e.g., “Emotional difficulties like many things, tend to work out by themselves”) on a 4-point Likert scale from 1 = “strongly disagree” to 4 = “strongly agree”. Internal reliability is very good (α = .84).

With an adapted version of the *ACMQ* [27] the motivation of subsequent help-seeking is assessed. Participants are asked to rate how much they agree with statements of why they might seek out help (e.g., “I could imagine seeking help for my current complaints, because I feel like I would personally benefit from it”) on a 5-point Likert scale from 1 = “strongly disagree” to 5 = “strongly agree”. Final reliability scores will be calculated with the data.

With a list of potential persons (e.g., psychotherapist) and institutions (e.g., counselling centre) where participants could seek help (adapted from [41]) help-seeking intention and behaviour is assessed on a 7-point Likert scale from 1 = “extremely unlikely” to 7 = “extremely likely”. This response format has previously been validated for general help-seeking [64]. This instrument will be used to test the hypotheses H1, H2, and H3.

With the *TPB* [60, 61] subjective norms (items 1 to 4), attitudes (items 5 to 7) and perceived control (items 8 to 10) of help-seeking, theoretically based on the Theory of Planned Behaviour [1], is assessed. All items measuring subjective norms (α = .84) and perceived behavioural control (α = .74) were rated on a 7-point Likert scale from 1 = “strongly disagree” to 7 = “strongly agree”. To measure attitudes (α = .69), participants indicated their attitude towards seeking professional help on three pairs of bipolar items (e.g., “good” – “bad”).

#### Stigmatising attitudes

Different instruments are used to assess a scope of stigmatising attitudes that theoretically influence the help-seeking process. These instruments and questions will be used to test the hypotheses H4, H5, H6, and H7.

With the *SSMIS-public* and *-self-SF* [12] perceived public stigma and the agreement thereof are assessed. Participants are asked to rate how much they agree with statements concerning people with a mental illness (e.g., “Most people with mental illness are dangerous”). The statements are primed with “I think the public believes …” (public-stigma) or “I think …” (self-stigma) and participants can rate their answers on a 5-point Likert scale from 1 = “strongly disagree” to 5 = “strongly agree”. Internal reliability for the public-stigma subscale (α = .73) and self-stigma subscale (α = .75) are very good.

With the *SDS* [2] the tendency of a person to distance themselves socially from people suffering from mental illnesses is assessed. Participants are asked to rate how much they agree with statements concerning people with a mental illness (e.g., “How willing would you be about renting a room in your home to a person with severe mental illness?”) on a 5-point Likert scale from 1 = “very unlikely” to 5 = “very likely”. Internal reliability is very good (α = .85).

With four items the agreement with the stereotype of blame is assessed [49]. Participants are asked to rate how much they agree with statements (e.g., “Persons with mental illness are to blame for their problems”) on a 5-point Likert scale from “1 = don’t agree at all” and “5 = agree completely”. Internal reliability is very good (α = .79).

With three items support for discrimination of people with a mental illness is assessed [49]. Participants are asked to rate how much they agree with statements (e.g., “If persons with mental illness do not consent to medical treatment, they should receive compulsory treatment”) on a 5-point Likert scale from 1 = “don’t agree at all” to 5 = “agree completely”. Internal reliability is good (α = .71).

With the *SSOSH-SF* [7] the proclivity of self-stigma for seeking out professional help for one’s mental health problems is assessed. Participants are asked to rate how much they agree with statements (e.g., “I would feel inadequate if I went to a therapist for psychological help”) on a 5-point Likert scale from 1 = “don’t agree at all” to 5 = “agree completely”. Internal reliability is excellent (α = .87).

With the *ERMIS* [4] the emotional reaction towards a person with a mental illness is assessed, with its subscales indicating pro-social reactions (e.g., “I feel pity towards the person”), fear (e.g., “The person makes me feel afraid”), and anger (e.g., “I react annoyed”) towards a person with a mental illness. Participants are asked to rate how much they agree with statements on a 5-point Likert scale from 1 = “don’t agree at all” to 5 = “agree completely”. Final reliability scores will be calculated with the data.

With two items shame experienced for being mentally ill (“Would you feel shame, if you had mental problems?”) and seeking out help (“Would you feel shame, if others knew that you were seeking out professional help for you mental problems?”) is assessed. Participants can rate their response with the options 1 = “not at all”, 2 = “a little”, 3 = “fairly”, 4 = “fairly strong”, and 5 = “very strong”. Final reliability scores will be calculated with the data.

#### Context assessment measures

With the *PHQ-9* [32] depressive symptoms are assessed. Participants are asked to rate how often they have been bothered by problems over the last 2 weeks (e.g., “Little interest or pleasure in doing things”) with the response options 1 = “not at all”, 2 = “several days”, 3 = “more than half the days”, 4 = “nearly every day”. Internal reliability (α = .89) and test-retest reliability after 48 h (*r* = .84) are excellent.

With the *GAD-7* [32] anxiety is assessed, seen as anxiety disorders are often co-morbid with depressive disorders. Participants are asked to rate how often they have been bothered by problems over the last 2 weeks (e.g., “Feeling anxious, nervous, or on edge”) with the response options 1 = “not at all”, 2 = “several days”, 3 = “more than half the days”, 4 = “nearly every day”. Internal reliability (α = .92) and test-retest reliability (*r* = .83) are excellent. Additionally, the open-ended question “Do you have further psychological complaints?” is asked, to control for other co-morbid symptoms.

With five consecutive questions [61] perceptions about context and infrastructure of help-seeking are assessed: (i) local service awareness: whether they knew where to find a professional in the vicinity (1 = “yes”, 2 = “no”), (ii) spatial distance: if so, how long it would take them to reach the professional (in minutes), (iii) accessibility: whether they believed they would get an appointment (1 = “yes”, 2 = “no”), and if so, (iv) temporal distance: when this appointment would take place (1 = “today”, 2 = “tomorrow”, 3 = “in ‘xx’ days”, 4 = “in ‘xx’ weeks”, 5 = “in ‘xx’ months”, with the option to enumerate “xx days/weeks/months,” (v) treatment efficacy beliefs: to what extent they consider it likely that they get appropriate help there for their current problems from 1 = “very unlikely” to 7 = “very likely”.

With three to seven questions (filtered, depending on previous answers) previous treatment experience (e.g., “Have you ever received treatment for mental illness in your life?”) and official diagnosis (“What official diagnosis did the doctor give you?”) are assessed. Additionally, treatment contentment was assessed with the question “How content were you with your psychiatric or psychotherapeutic treatment in general?” to which the participants can respond on a 5-point Likert scale from 1 = “very discontent” to 5 = “content”. These questions will be used to test the hypotheses H3 and H7.

Finally, questions were formulated to assess the adherence to (“To what extent do you adhere to the measures to contain the coronavirus?”), felt restriction of (“How much do you feel restricted by the measures?”), and felt mental burden of the lockdown restrictions (“How much psychological strain do the measures put on you?”) with respective 5-point Likert response scales. The perceived possibility to go to a general practitioner or therapist is assessed: “Despite the current corona pandemic, would you go to the doctor or therapist because of your psychological complaints?” and participants can respond 1 = “Yes, I would go anyway”, 2 = “No, I wouldn’t go at the moment because of Corona”, and 3 = “No, I wouldn’t go anyway”.

### Data management

Data of all assessment times and of the study intervention will be documented. Plausibility checks will be carried out as soon as the data is entered into the system. Data will be stored for 10 years in a central study database based on the data management system of the Institute of Psychology of the University of Greifswald according to current standards of data security laws formulated in the General Data Protection Regulation of 2018. Data collection will happen pseudonymously, with person-identifying data and experimental data being stored separately. Scientific researchers will not get access to person-identifying data, as it is stored by the online-panel “respondi AG”. Reversely, “respondi AG” will not have access to experimental data.

Participants included in the study give electronic informed consent following comprehensive written information about the study. If a participant limits given consent, the restriction is documented and further data assessment is stopped according to the specified restrictions. If a participant withdraws the given consent at any stage of the study, all data is removed, excluding data published prior to the withdrawal. Data will be exclusively analysed anonymously and publications will use aggregated data not allowing for personal identification. If data is shared it will only be shared without participant’s identifiers.

#### Data collection & monitoring

A data monitoring committee (DMC) was not installed, as there is no blinding to experimental manipulations and adverse events can directly be reported to those responsible for the study. After a preliminary “soft-launch” of *N* = 100 data quality will be analysed and technical functioning checked. All outcomes will be analysed pseudonymously following data collection.

#### Harms

No harm is anticipated since the participation is voluntary and the intervention material is non-invasive. The affiliated ethics board has approved the study. Furthermore, the intervention was received positively during the cognitive debriefings by people suffering from depression (*N* = 15), indicating feasibility and harmlessness.

Nevertheless, to meet any acute situation of suicidality an informational text is shown to all participants who will be indicating thoughts of self-harm or suicide. In case the last PHQ-9 item (“Thoughts that you would be better off dead, or of hurting yourself”) scored “several days” or higher the participants will be informed of possible contact points and phone numbers they could call in the case of acute suicidal thoughts and intentions, including the German emergency ambulance number 112. Additionally, the study information includes contact details of two scientific researchers (LJP and TM) with explicit instruction to contact them at any time.

### Statistical methods

To answer the various research questions, the following statistical methods will be used: correlation analyses, (OLS-) regression analyses, multivariate analysis of variance with repeated measurements, multilevel analysis and Chi-square tests. Path models will be used to analyse the extended help seeking process-model. Appropriate statistical software (IBM SPSS 27, IBM AMOS, Stata, Mplus) will be used for all statistical analyses.

## Discussion

The lack of health care utilisation amongst people suffering from depression [6, 63] and the stigma attached to having a mental illness [11, 29, 45] are two societal issues that have to be addressed. Anticipated discrimination has an adverse impact on the lives of most people with depression [34] and even though there have been changes in the past few decades, with higher societal acceptance of people with a mental illness and better legal regulations, stigmatisation and fear of stigmatisation is still a significant barrier for people with a mental illness when seeking professional help [10]. Many web-based interventions, psychological self-tests, and nation-wide prevention campaigns already exist to tackle this problem and yet they are often not associated with increased health care utilisation due to their lack of addressing the behavioural component [9]. Another risk is that they can cause reactance amongst people already suffering from depressive complaints [56].

This project takes a step back and regards the variables inhibiting and encouraging mental health care utilisation. These proposed variables interact in a complex model of help-seeking. The intermediary variables that will be manipulated during this study were decided upon based on the results of a previous project, which analysed the importance of stigmatising attitudes within the help-seeking process for people with untreated mental illnesses in the general population.

On the basis of the expected results of the planned project, promising research and intervention perspectives can be developed: First, the project will provide a more detailed empirical investigation and conceptualisation of the stages of mental health care utilisation, as well as an examination of theoretical approaches to stigmatisation. Second, the results of our online study could highlight points of interest for an evidence-based design and evaluation of online interventions for mental illnesses, for which there exists an urgent need in the face of the increasing abundance of eclectic, unscientific, and commercially oriented online offers and applications. This need is driven by the ongoing virtualisation of professional health care, especially under developments of the Covid-19 pandemic.

### Limitations and generalisability

Our study design has a few limitations. Firstly, since one of the aims of the study is to find points of interests for evidence-based design and evaluation of online offers, the setting has to be online. Online studies have the problem of non-accountability. The attention of the participants is not controlled for, they can take unnecessary and lengthy breaks and it is not possible to track what they might do while participating, impeding data quality. Inherent to an online setting, the anticipated drop-out rate is substantial between the first baseline-assessment and the follow-up assessments and it is difficult to ensure study adherence over such a long period of time. This said, the online setting can be considered a strength since the targeted group is people with depression, who might be more willing to participate in a low-threshold online study than a full clinical trial. Secondly, the intervention is short and informational, without sufficient material to reflect upon one’s own problems extensively. The intervention, furthermore, is administered only once. Both these limitations will probably mean small effect sizes. Thirdly, only self-report measures can be used, which are inherently subjective and prone to perception biases. Considering the difficult subject matter social desirability might be an issue though online-anonymity should counteract this bias. Fourthly, the participants will have to be able to access electronic devices, wherefore there will most likely be a bias in our study population of people who are more technically proficient than others (especially in the age group 65+). Even though these limitations mean a reduced internal validity they reflect the real-life, online setting, therefore, external validity of the study will be high. A further limitation is the recruitment method. Participants will be recruited through an online-panel. Consequently, they have had to have signed-up previously and probably are part of a highly motivated sub-population, even though they all have a PHQ-9 score of at least eight indicating mild depression or higher. They are also used to answering questionnaires quickly, which might reduce data quality, since they will be aware of questionnaire internal attention checks.

## Conclusion

In sum and despite of these limitations, our study has the potential to discover points of interests and underlying mechanisms in a comprehensive process starting with the subjective sense of illness, over a self-identification as mentally ill, and in the best of cases ending in help-seeking behaviour.

## Supplementary Information

**Additional file 1.**

## Data Availability

The study results will be released to the scientific public by conferences and international peer-reviewed impact journals as well as to the general public by presentations in health care organizations and national congresses. The datasets generated during the current study are available from the corresponding author on reasonable request and will be made available in a scientific repository.

## Abbreviations

ACMQ: Autonomous and controlled motivation questionnaire
ATSPPH-SF: Short-form of the attitudes towards seeking professional help questionnaire
BRAHMS: Berlin risk appraisal and health motivation study, 1996
B-IPQ-R: Brief revised illness perception questionnaire
CSM: Common-sense modell of self-regulation
DFG: Deutsche Forschungsgemeinschaft (i.e., German Research Foundation)
ERMIS: Emotional reactions to mental illness scale
EUROHIS-QoL Index: Short-form of the World Health Organisation quality of life instrument
GAD-7: Generalized anxiety disorder (PHQ-anxiety sub-scale)
PHQ-9: Patient health questionnaire (depression sub-scale)
SDS: Social distance scale
SELF-I: Self-identification as mentally ill scale
SF-1: Short-form of the general health questionnaire
SSMI-public & -self-SF: Short-form of the self-stigma of mental illness questionnaire
SSOSH-SF: Short-form of the self-stigma for seeking help questionnaire
TPB: Theory of planned behaviour
