# Supplementary material for: How can the utilisation of help for mental disorders be improved? A quasi-experimental online study on the changeability of stigmatising attitudes and intermediate variables in the process of utilisation
Source: BMC Public Health. 2021 Nov 19;21:2124. doi: 10.1186/s12889-021-12125-5 (PMC8602987; doi:10.1186/s12889-021-12125-5)
Supplement: Supplementary file 1 — Additional file 1. [file 12889_2021_12125_MOESM1_ESM.docx]

**Online supplementary material for the following article:**

McLaren, T^[[1]](#footnote-1)^., Peter, L.-J., Tomczyk, S., Muehlan, H., Stolzenburg, S., Schomerus, G., Schmidt, S. (2021). How can the utilisation of help for mental disorders be improved? – Study Protocol: A quasi-experimental online study on the changeability of stigmatising attitudes and intermediate variables in the process of utilisation. *Paper*, *Vol.*(x), pp-pp. doi.

**Intervention Structure**


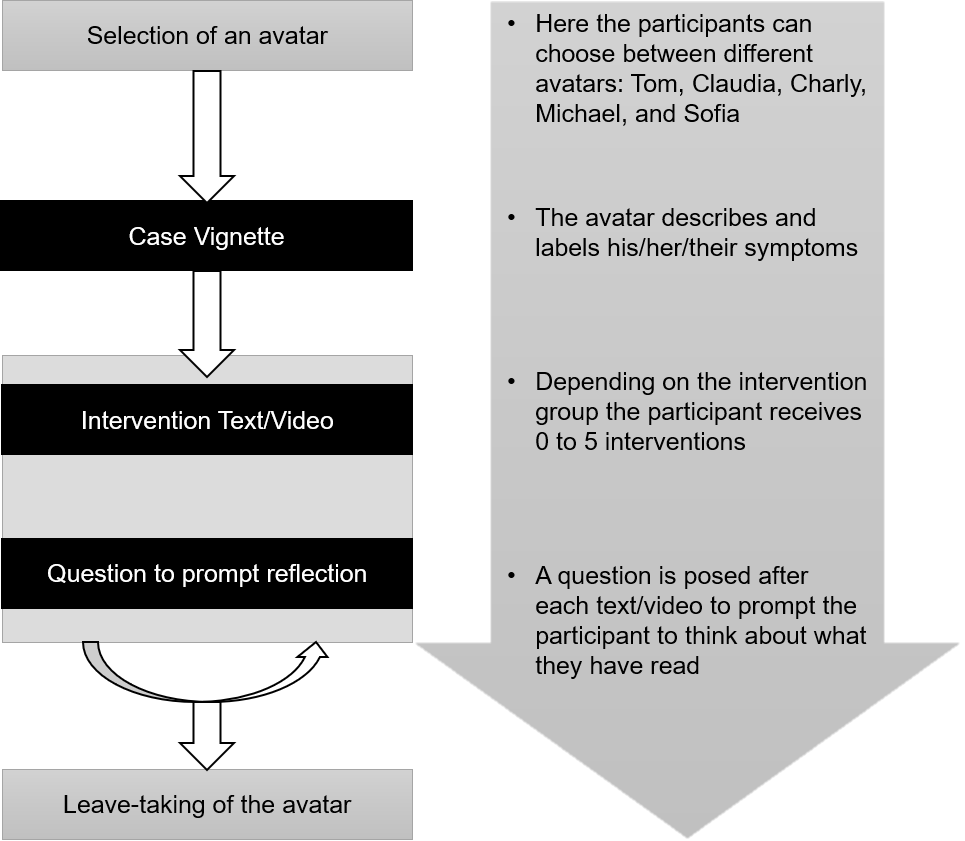


**Intervention Material**

***Avatar selection***

On the following pages, a person will tell you about their personal experiences dealing with their own psychological complaints.

For this purpose, please choose a person. It’s up to you which person you choose, for example, a person who appears likeable to you or with whom you can identify.

***Case Vignette***

Hello,

I’m NAME and I’d like to tell you about my recent experiences. For a few months now I’ve often felt sad and can’t even explain what’s been pulling me down. It’s been really frustrating!

I rarely experience joy anymore and spending time with my friends isn’t as fun as it used to be. On some days I already struggle with minor decisions in the morning, such as choosing what to wear or what I’ll have for breakfast.

I find it hard to pull myself together to leave the house and I have trouble concentrating on my work. I often find myself worrying extensively about little things and I blame myself for everything and anything. I sometimes struggle with myself, even though I have come to realise that these are signs of depression.

***Intervention: Introduction***

You’ve already gotten to know NAME a little bit and might be able to understand her/him/them.

NAME has followed up on his/her/their depression. Some realisations have given him/her/them courage and helped her/him/them so much that he/she/them would like to share these with you.

On the following pages you will gain insight into NAME’s personal experiences and thoughts.

You will repeatedly have the opportunity to reflect on what you might be able to take away from NAME’s experiences. We would like to encourage you to think about which of NAMES’ experiences might be particularly relevant for you personally.

***Intermediary Variables – All Texts & Questions to prompt reflection***

Continuum Beliefs

I wondered: Am I different now? Am I not normal anymore? Of course, everyone experiences negative feelings and internal stress from time to time, especially in difficult situations. The question, rather, is how long those negative feelings last for and how much of a burden they are. Sometimes, when I feel particularly bad I forget that I’ve ever felt better and I can’t imagine feeling good in the future.

I find it helpful to think of a scale all people are located on. Nobody is 100% healthy or sick, everyone is situated somewhere in between. There isn’t an all or nothing, no “normal” or “abnormal”, but rather fluid transitions. A depression can change and is treatable.

However, to support people with these changes, it’s important to have descriptions for this condition. To label the condition as “depression” helps to identify and treat it.

*…Continuum Beliefs – question*

Take a moment to think about what you just read/saw.

How would you describe your mental health right now? Please move the slider to the fitting position.

1 2 3 4 5 6 7

(I’m currently feeling sick) (I’m currently feeling healthy)

Causal Beliefs

Sometimes, I wonder why I’ve got a depression. After all, I’ve been through various crises before. So, I started to search for possible causes – and was surprised of how diverse they can be. Causes can be of a physical or mental nature, or be situated in the environment. Some examples are experiences such as loss, childhood upbringing, one’s ways of thinking or dealing with emotions.

In an interview with a psychologist I heard that one can picture the psyche as a cup. Depressive complaints develop when the cup is too full and overflows. What the cup is filled with can be very different. For example, a family disposition can form a “sediment”, so that less space remains in the cup to begin with. When the cup is filled to the top, an additional crisis can eventually cause “the cup to overflow”^[[2]](#footnote-2)^. In this case, one’s own resources might not be sufficient to handle the additional stress. The exact trigger might not even be obvious.

*…Causal Attribution – question*

Take a moment to think about what you just read.

How full does your inner “burden-cup” feel? Please move the slider to the fitting position.

1 2 3 4 5 6 7

(Currently too full) (Currently pretty empty)

Mental Health Literacy

I wanted to find out as much as possible about Depression. There is a lot of information on the internet. Nearly every fifth person suffers from depression once in their life. At first, most people go to the doctors because of physical complaints. Nobody is completely protected but the risk is lower, if you have support, e. g. from friends or family.

I learned that depression can manifest itself and progresses in different ways. Then I thought: „sure, back pain can be different, too – some feel tension or neck pain and others have a slipped disc”. In reports about their help-seeking many affected people talked about their fear of being a failure. I noticed that I too, had negative thoughts. But there were also positive examples.

A depression can be treated by talking to a psychotherapist, medication or a combination of both. Most of the people report that treatment has helped in the long run, although it is not an easy time. Many report that they have learned things in psychotherapy that help them later in life to cope better despite crises.

…Mental Health Literacy – question

Take a moment to think about what you just read.

Do you want to find out more about depression for yourself? Please move the slider to the fitting position.

1 2 3 4 5 6 7

(I don’t want to find out (I want to find out

more about depression) more about depression)

Self-Efficacy (Self-Help)

I wanted to change something about my life and my habits despite my stressful daily routine. It really did help me, although sometimes, I was close to quitting and often didn‘t manage. I got used to writing down how I’m doing and what I‘ve been thinking about every day. This ritual helps me structure my feelings and thoughts.

Every Sunday I write a weekly schedule. For each day, I write down what I have to do and what I want to do. The plan is just a support, so I take it easy. Even tough, I sometimes have trouble getting out of bed.

Then I use my „joy lottery“, which is a jar full of slips of paper with activities that I‘ve enjoyed. I draw a slip of paper and do what it says – whether I want to or not. For example: “call a friend”. He doesn’t always answer, but that’s okay. Also, every day I go for a walk in the park, especially then, when I don’t feel like it. Rainy weather is no longer an excuse

…Self-Efficacy (Self-Help) – question

Take a moment to think about what you just read.

To what extent can you imagine using one/several of the self-help strategies as described in your everyday life? Please move the slider to the fitting position.

1 2 3 4 5 6 7

(I can’t imagine it) (I can imagine it very well)

Self-Efficacy (Utilisation of Professional Help)

After a lot of back and forth, I decided to seek professional help. I knew it would be a challenge, but I wanted change and my friends encouraged me with their experiences.

First, I searched on the internet for possible therapies – there are promising options. Before my doctor’s appointment I wrote down my problem. I thought: “maybe I won’t be able to say what‘s wrong or I am to ashamed and then I’ll just show my notes.” At first I had a hard time opening up, but my doctor listened and then inquired further. She referred me to a psychotherapist and prescribed an antidepressant. I struggled a lot to take it, but I tolerate it well.

I wasn’t prepared for how long I had to wait for an appointment with the therapist but I stuck to my plan. When therapy started, I thought, “everything will get better.” However, some sessions were very tough! Still, I am happy I found my therapist and now I understand that depression is treatable

…Self-Efficacy (Utilisation of Professional Help) – question

Take a moment to think about what you just read/saw.

What do you think about the decision to seek professional help? Please move the slider to the fitting position.

1 2 3 4 5 6 7

(I would do it differently) (I would do it exactly the same)

***Intervention: End (leave-taking of the avatar)***

I don’t know if you are in a similar situation – if so, I can tell you: I know exactly how hard it is! Maybe it helped you to read about my experiences.

Best regards and all the best for you,

NAME

1. Corresponding author: Thomas McLaren, Department of Health and Prevention, Institute of Psychology, University of Greifswald, Robert-Blum Str. 13, 17489 Greifswald, Germany

   Tel.: +49- 3834- 420- 3814

   Mail: thomas.mclaren@uni-greifswald.de [↑](#footnote-ref-1)
2. Here a well-known German saying is used: “Der Tropfen, der das Fass zum Überlaufen bringt”. The English equivalent is “the last straw that breaks the camel's back”. This doesn’t translate well, since an “overflowing cup” is usually connoted positively with wealth and prosperity. [↑](#footnote-ref-2)
